# Hydrothermally synthesized nanostructured LiMn_x_Fe_1−x_PO_4_ (*x* = 0–0.3) cathode materials with enhanced properties for lithium-ion batteries

**DOI:** 10.1038/s41598-021-91881-1

**Published:** 2021-06-10

**Authors:** Dung V. Trinh, Mai T. T. Nguyen, Hue T. M. Dang, Dung T. Dang, Hang T. T. Le, Huynh T. N. Le, Hoang V. Tran, Chinh D. Huynh

**Affiliations:** 1 School of Chemical Engineering, Hanoi University of Science and Technology, 1st Dai Co Viet Road, Hanoi, Vietnam; 2 Faculty of Chemistry, VNUHCM-University of Science, 227 Nguyen Van Cu Street, Ho Chi Minh City, Vietnam

**Keywords:** Chemistry, Materials science, Physics

## Abstract

Nanostructured cathode materials based on Mn-doped olivine LiMn_x_Fe_1−x_PO_4_ (*x* = 0, 0.1, 0.2, and 0.3) were successfully synthesized via a hydrothermal route. The field-emission scanning electron microscopy (SEM) and energy-dispersive X-ray spectroscopy (EDS) analyzed results indicated that the synthesized LiMn_x_Fe_1−x_PO_4_ (*x* = 0, 0.1, 0.2, and 0.3) samples possessed a sphere-like nanostructure and a relatively homogeneous size distribution in the range of 100–200 nm. Electrochemical experiments and analysis showed that the Mn doping increased the redox potential and boosted the capacity. While the undoped olivine (LiFePO_4_) had a capacity of 169 mAh g^−1^ with a slight reduction (10%) in the initial capacity after 50 cycles (150 mAh g^−1^), the Mn-doped olivine samples (LiMn_x_Fe_1−x_PO_4_) demonstrated reliable cycling tests with negligible capacity loss, reaching 151, 147, and 157 mAh g^−1^ for *x* = 0.1, 0.2, and 0.3, respectively. The results from electrochemical impedance spectroscopy (EIS) accompanied by the galvanostatic intermittent titration technique (GITT) have resulted that the Mn substitution for Fe promoted the charge transfer process and hence the rapid Li transport. These findings indicate that the LiMn_x_Fe_1−x_PO_4_ nanostructures are promising cathode materials for lithium ion battery applications.

## Introduction

The production and storage of new and clean energy are two major challenges that face humanity to stop the depletion of natural resources. In modern life, a wide range of systems such as portable electronic devices, computers, phones, cameras, and electric vehicles require stable and safe electrical energy. Such diversity in the electronics market compels the development of different electrochemical systems such as non-flammable batteries, superconductors, and other types of batteries^1–5^. Since 1970, the phosphorus-olivine structure has been tapped as a potential material for Li-ion batteries^6–10^. In particular, lithium iron phosphate (LiFePO_4_) and lithium manganese phosphate (LiMnPO_4_) are some of the most studied among transition metal oxide cathode materials due to their high theoretical capacity (~ 170 mAh g^−1^), inherent chemical stability, increased safety due to their lower explosion risk from overcharging, better thermal stability, and lower material cost^11–14^. However, the electronic conductivity (δ) and Li-ion diffusion coefficient (D_Li_) of LiFePO_4_ and LiMnPO_4_ are on the low end; for example, δ_LiFePO4_ = 10^–8^–10^–9^ S cm^−1^ and δ_LiMnPO4_ = 10^–10^ S cm^−1^^12,14–16^. Due to the disadvantage of these materials’ conductivity on electronics and ion transport, there exists a major constraint to commercial applications of these materials^17,18^. To mitigate these issues, three approaches are typically considered: (i) coating carbon on the surface of olivine to improve the electron transfer process^9,12,19–22^; or (ii) fully or partly substituting of Fe by a 3d-transition metals (such as Mn, Ni or Co)^8,9,18,23–26^. Previous works reported that cation mixing in LiMn_x_Fe_1−x_PO_4_ orthophosphates is a great trade-off between the valuable capacity of LiFePO_4_ and the high potential of LiMnPO_4_ (voltage of oxidation pair compared with Li^+^/Li)^27–29^. These studies also shown that doping transition metal cations into LiFePO_4_ or LiMnPO_4_ causes a narrowing of the energy gap, which may improve the electrical conductivity of this material^3,29^. (iii) The Li-ion diffusion coefficient (D_Li_) of olivine cathode materials also can be improved by reducing the particle size of the olivines to nanoscale, or changing their particle shapes to nanorod, nanoplates or nanorectangular sheets^1,13,20,27,30–32^. In addition, the electrochemical efficiency in olivine phosphate cathode materials also strongly depend on the prepared techniques solid-state reactions^1,33^, sol–gel methods^13,34^, reactions from hydrothermal routes^11,24,31,32,35–38^ or microwave plasma chemical vapor deposition^19^. Among these methods, the hydrothermal synthesis is a facile water-based precipitation technique that enables the control of the nucleation and development of the crystal.

In this work, we improve the chemical properties by partly substation Fe by Mn, nanosizing of particles size and carbon coating of the original olivine. In which, the nanostructured of Mn doped olivine LiMn_x_Fe_1−x_PO_4_ (x = 0, 0.1, 0.2, and 0.3) were synthesized by a facile hydrothermal route, and the as-prepared olivines were coated with carbon through pyrolysis. Electrochemical studies showed that the olivines were capable of delivering a reversible capacity around 160 mAh g^−1^ at a current density of C/10 in the voltage range of 2.5–4.3 V (vs. Li^+^/Li). The improving on the kinetics of Li transport in these olivines were found and also discussed.

## Experimental

The chemicals Mn(NO_3_)_2_ (≥ 99 wt%), FeSO_4_·7H_2_O (≥ 99 wt%), LiOH (≥ 98 wt%), NH_3_ solution (30 wt%), H_3_PO_4_ (≥ 85%), citric acid monohydrate (C_6_H_8_O_7_·H_2_O, ≥ 99.5 wt%), polyvinylidene fluoride (PVDF), ethylene carbonate (EC, 98 wt%), and dimethyl carbonate (DMC, ≥ 99 wt%) were purchased from Sigma-Aldrich. All the other chemicals were of analytical grade and used without further purification. All the aqueous solutions were prepared with deionized water (DI water, 18.2 MΩ.cm) for all the experiments.

### Synthesis of olivine samples

A mixture of H_3_PO_4_, Mn(NO_3_)_2_, FeSO_4_·7H_2_O, and C_6_H_8_O_7_·H_2_O were dissolved into 50 mL DI water under N_2_ atmosphere at 1:9, 2:8, and 3:7 molar ratios of Mn:Fe. Then, 20 mL of 0.5 M LiOH was dropped into the mixture at a 3:1 ratio of Li^+^:PO_4_^3−^. The NH_3_ solution was dropped into the final mixture to adjust the pH to 6–6.5. The final mixture was stirred for 30 min at 60–80 °C to form the sol. The sol was transferred to the autoclave at 180 °C for 12 h. The precipitation was collected by filtering, washing, and drying at 95 °C for 24 h.

### Characterizations and methods

The X-ray diffraction spectra were recorded on a D5005-SIEMENS diffractometer with Cu-Kα radiation (λ = 1.54056 Å). The synthesized olivines were observed using a Hitachi FE-SEM S-4800 field-emission scanning electron microscope coupled with an Oxford 300 EDX analysis system. The micro-Raman spectra were recorded on a LABRAM-1B spectrophotometer (Jobin Yvon, France). The electrochemical properties of the synthesized olivines were evaluated by cyclic voltammetry (CV) at a scan rate of 1 mV s^−1^, galvanostatic charge/discharge tests, and electrochemical impedance spectroscopy by using a VMP3 apparatus (BioLogic, France) in the frequency range 5 mHz to 105 Hz and 10 mV peak-to-peak excitation signal.

### Devices preparation

The synthesized olivine powders were mixed with carbon black as a conductive agent, polyvinylidene fluoride (PVDF) as binder in the ratio of 90:5:5, respectively, in a porcelain mortar and pestle. The prepared mixtures were coated onto a 0.1 mm aluminium sheet, dried at 120 °C in an oven for 12 h, and pressed on a press machine to create a thickness of 20 μm. The electrodes were then formed to circular plates with a diameter of 1.6 cm on a stamping machine. Coin-cells CR-2032 were assembled in an argon-filled glove box with an anode of lithium metal. The electrolyte solutions were 1 M LiPF_6_ in a mixture of ethylene carbonate (EC) and dimethyl carbonate (DMC) at a volumetric ratio of 2:1.

## Results and discussion

### Structural and morphological characterizations

**Figure 1:**
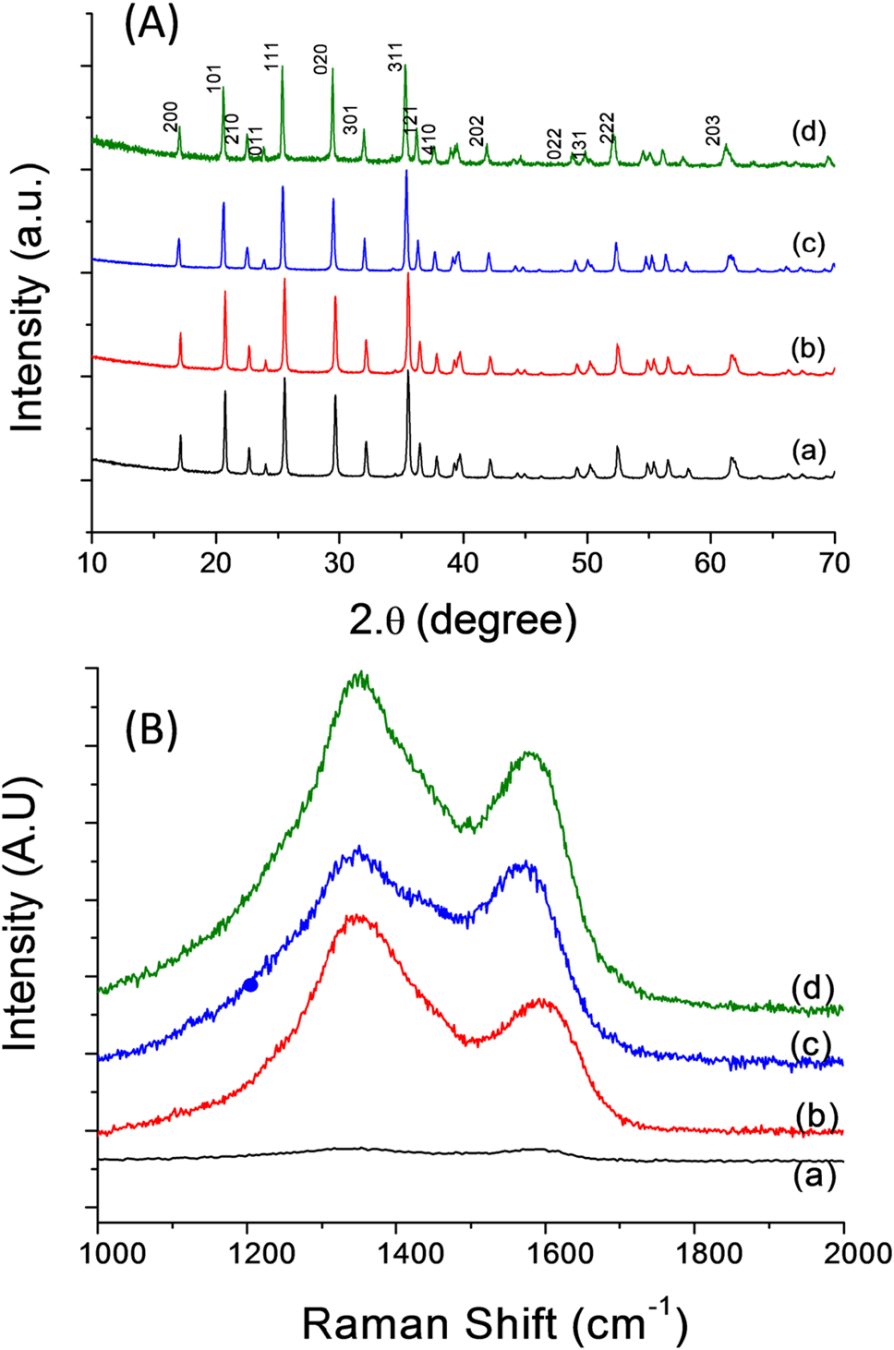
(**A**) X-ray diffraction patterns and (**B**) Raman spectra of olivine samples: (a) LiFePO_4_; (b) LiMn_0.1_Fe_0.9_PO_4_; (c) LiMn_0.2_Fe_0.9_PO_4_ and (d) LiMn_0.3_Fe_0.7_PO_4_, respectively. These figures have been presented using OriginLab Software, ver.6.0 (https://www.originlab.com).

The XRD spectra of the synthesized olivine samples (Fig. 1A) show a pure and very well crystallized single olivine phase without impurity (e.g., MnO_2_, Li_2_O, and Fe_2_O_3_). All the diffraction peaks were identified in an orthorhombic structure with a space group of P_nma_ (JCPDS 81-1173)^14,27^. In the olivine phase, the atoms are located at 4a for lithium, 4c for iron, 4c for phosphorous, and 4c and 8a for oxygen. The Mn substitute of Fe is at the 4c site. The lattice parameters are calculated by the Celref program and detailed in Table 1. The lattice expansion of the Mn-doped olivine samples depended on the degree of Mn substitution as well as the larger ionic radius of Mn^2+^ (r_Mn2+_ = 0.08 nm > r_Fe2+_ = 0.074 nm)^14,27^.

**Table 1:** Lattice parameters of Mn-doped olivine samples.

Samples	a (Å)	b (Å)	c (Å)	V (Å^3^)	Average crystalline size (nm)
LiFePO_4_	10.342	6.021	4.699	292.60	19.5
LiMn_0.1_Fe_0.9_PO_4_	10.330	6.012	4.702	292.01	22.5
LiMn_0.2_Fe_0.8_PO_4_	10.374	6.038	4.711	295.09	21.4
LiMn_0.3_Fe_0.7_PO_4_	10.390	6.048	4.718	296.47	21.0

**Figure 2:**
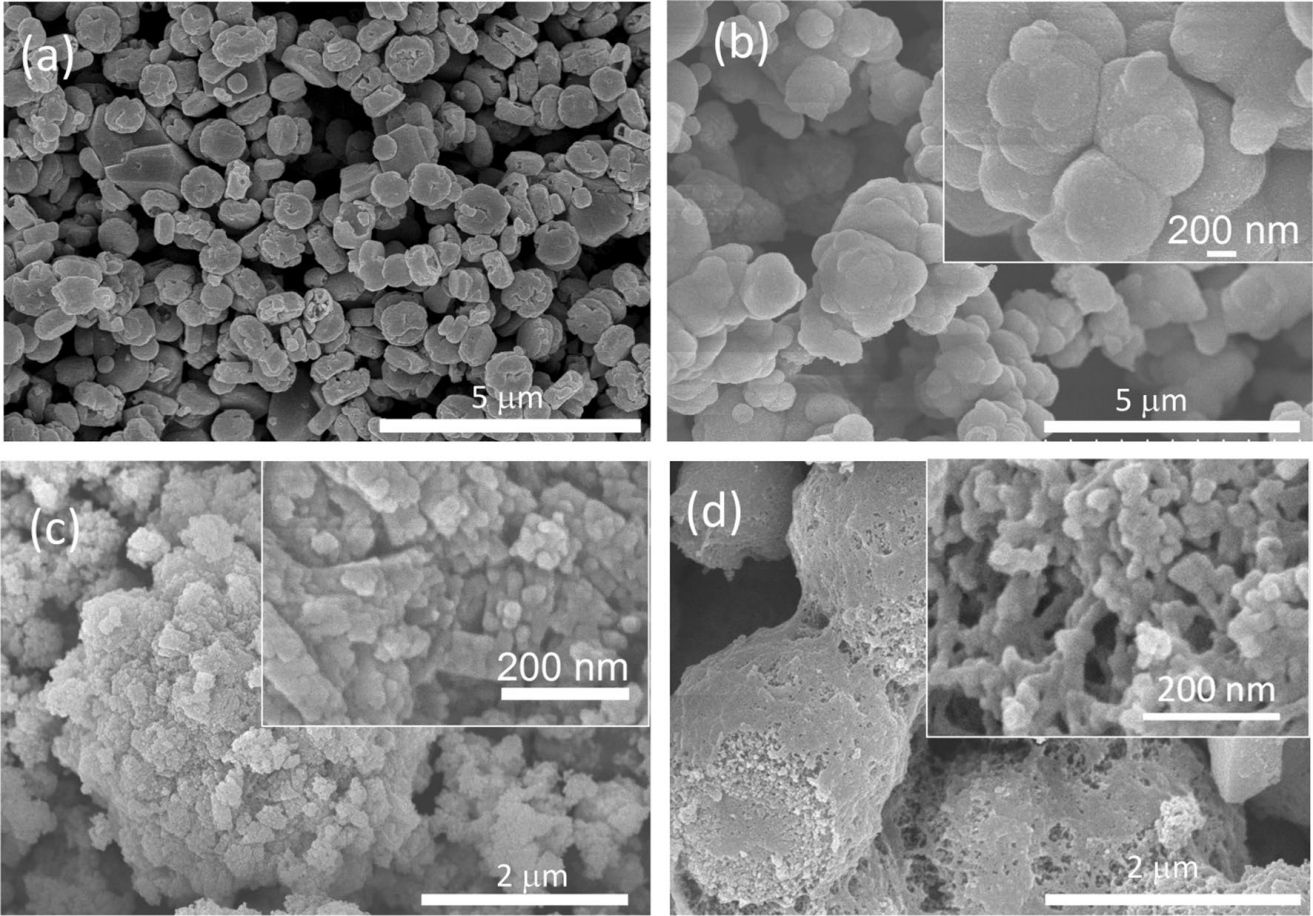
SEM images of synthesized olivine samples: (**a**) LiFePO_4_; (**b**) LiMn_0.1_Fe_0.9_PO_4_; (**c**) LiMn_0.2_Fe_0.8_PO_4_ and (**d**) LiMn_0.3_Fe_0.7_PO_4_.

**Figure 3:**
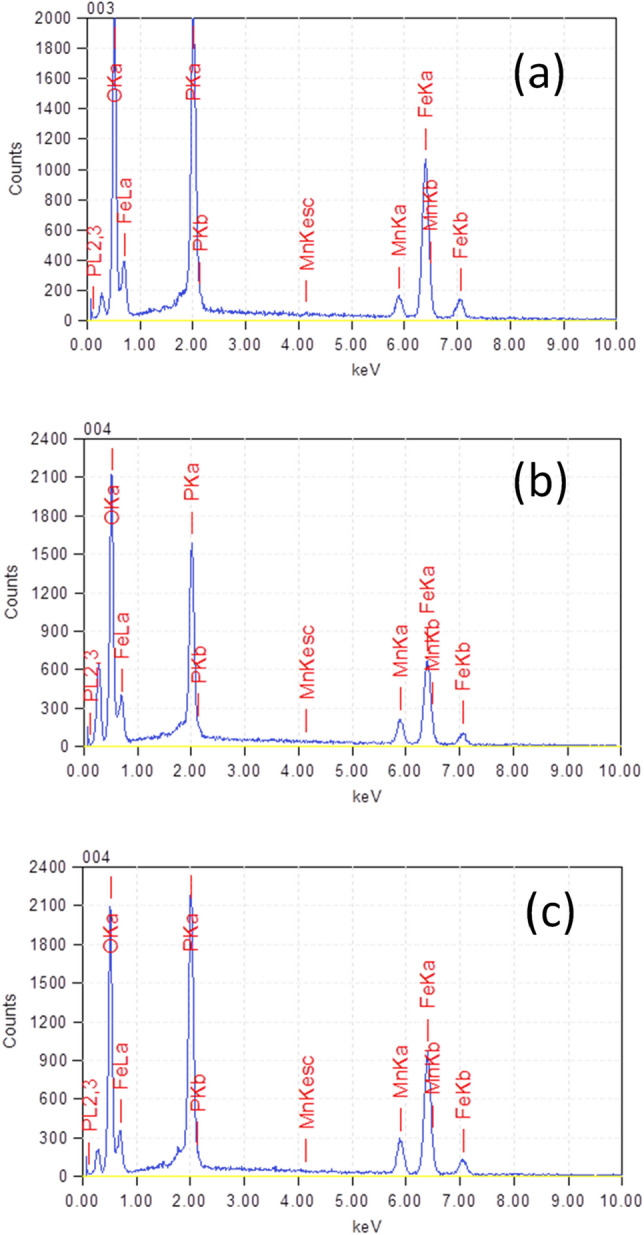
EDS spectra of Mn-doped olivine samples (**a**) LiMn_0.1_Fe_0.9_PO_4_; (**b**) LiMn_0.2_Fe_0.8_PO_4_ and (**c**) LiMn_0.3_Fe_0.7_PO_4_.

The broadening of the diffraction peaks was attributed to the nanocrystallinity of the samples. The average crystalline size is calculated from the full width at half maximum (FWHM) through the Debye–Scherrer equation^32^:

1 $$ d_{hkl} = \frac{k \cdot \lambda }{{\beta \cdot cos\theta }}, $$

where d_hkl_ is the average particle size, *k* is the constant depending on the crystallite shape (0.9), λ is the wavelength of the copper K_α_ X-ray radiation, β is the FWHM of the most intense peak (in rad), and θ is the diffraction angle. The crystallite size average can be estimated around 20 nm for all samples. This sub-micron size could indicate fast kinetics of lithium insertion due to a shortening of the lithium pathway for diffusion. The Raman spectra of three samples LiMn_0.1_Fe_0.9_PO_4_, LiMn_0.2_Fe_0.8_PO_4_ and LiMn_0.3_Fe_0.7_PO_4_ at high frequency 1000–2000 cm^−1^ (Fig. 1B) confirms the successful carbon coating on surface olivines from the pyrolysis process. The Raman spectrum showed the finger-print band of coated carbon through two characteristic peaks at 1351 cm^−1^ (D-band) and 1570 cm^−1^ (G-band), respectively. This result confirm the successfully coated carbon onto olivine surface^22,39^. The SEM image in Fig. 2 shows that the synthesized olivine LiMn_x_Fe_1−x_PO_4_ samples crystallized with uniform size. The SEM of the original olivine LiFePO_4_ sample (Fig. 2a) shows LiFePO_4_ as consisting of spherical-like particles with a narrow particle size distribution in range from 200 to 400 nm. When Mn is doped into LiMn_x_Fe_1−x_PO_4_, the particle size strongly reduces and the samples became more porous see Fig. 2b–d. It can be seen that particle size decreased with increasing Mn. In particular, the particle size of LiMn_0.3_Fe_0_._7_PO_4_ is around 50 nm (Fig. 2d). The EDX spectra of the three Mn-doped samples (Fig. 3) showed the indicators of Mn, Fe, P, and O and confirmed the stoichiometric relationship between Mn:Fe (Table 2) was similar to the desired composition.

**Table 2:** Elemental composition of the Mn-doped olivines determined by SEM–EDX.

Elements	keV	LiMn_0.1_Fe_0.9_PO_4_		LiMn_0.2_Fe_0.8_PO_4_		LiMn_0.3_Fe_0.7_PO_4_	
		Atom%	Mn/Fe	Atom%	Mn/Fe	Atom%	Mn/Fe
Oxygen	0.525	63.58	0.12:0.88	62.34	0.19:0.81	64.79	0.26:0.72
Phosphorus	2.013	16.11		17.20		14.03	
Manganese	5.894	2.46		3.84		5.55	
Ferrous	6.398	17.85		16.61		15.63	

### Electrochemical measurements

**Figure 4:**
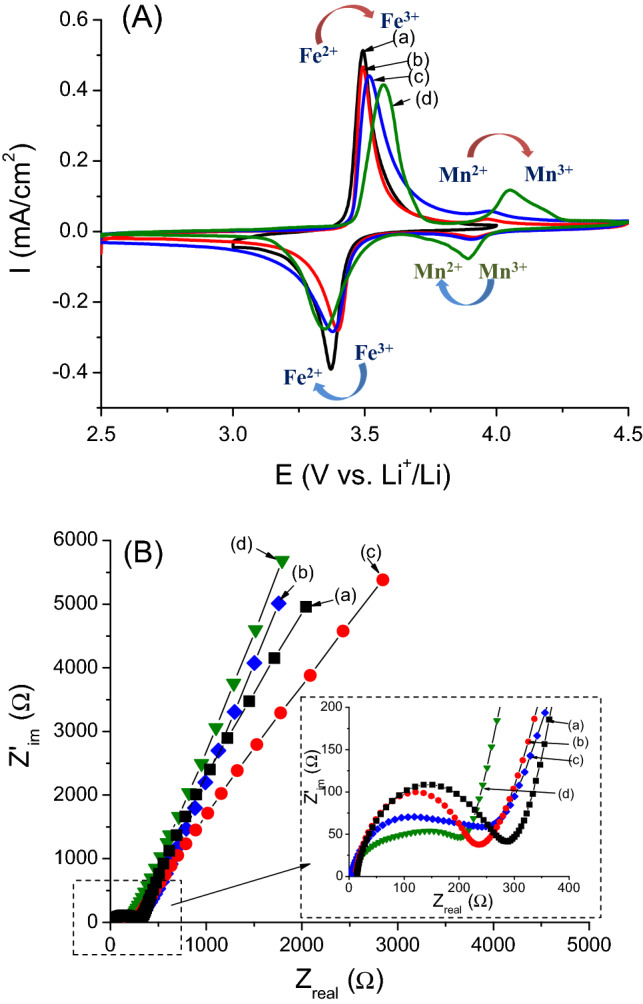
(**A**) Cyclic voltammograms at a scan rate of 5 mV s^−1^ and (**B**) corresponding Nyquist plots of electrochemical impedance spectroscopy (EIS) of synthesized olivine samples: (a) LiFePO_4_; (b) LiMn_0.1_Fe_0.9_PO_4_; (c) LiMn_0.2_Fe_0.8_PO_4_ and (d) LiMn_0.3_Fe_0.7_PO_4_. Experimental conditions are described in the text. These figures have been presented using OriginLab Software, ver.6.0 (https://www.originlab.com).

The CVs of the LiMn_x_Fe_1−x_PO_4_ (x = 0, 0.1, 0.2, and 0.3) samples at a scan rate of 1 mV s^−1^ are shown in Fig. 4A. The original olivine LiFePO_4_ was measured over a voltage range of 3–4 V while the Mn-doped olivines were scanned at a wider range of 2.5–4.5 V. As can be seen from the CVs, the traditional reversible peak of the Fe^3+^/Fe^2+^ couple at 3.45–3.55 V appeared in the obtained voltammograms. With a greater degree of Mn substitution, the redox peak in the high voltage region (~ 4 V) becomes more observable. On the other hand, Mn doping into the original olivine can also lead to a slight shift in the Fe-redox potential, which will be discussed further in cycling test section. The Nyquist plots of the EIS for the LiMn_x_Fe_1−x_PO_4_ (x = 0, 0.1, 0.2, and 0.3) samples (Fig. 4B) exhibited three regions: (i) a “quasi” semi-circle in the high-medium frequency corresponding to the charge-transfer; (ii) a straight line 45° from the real axis in low frequency region (in frequency range from 1 Hz to 0.1 Hz) demonstrating the Warburg impedance, and (iii) at very low frequencies (*f* < 10^–3^ Hz) the phase angle is increases due to the finite diffusion process^5,40^. It is observed that Mn doping is helpful for decreasing charge transfer resistance in olivine, which suggests fast electron transfer as well as stability in cycling performance.

**Figure 5:**
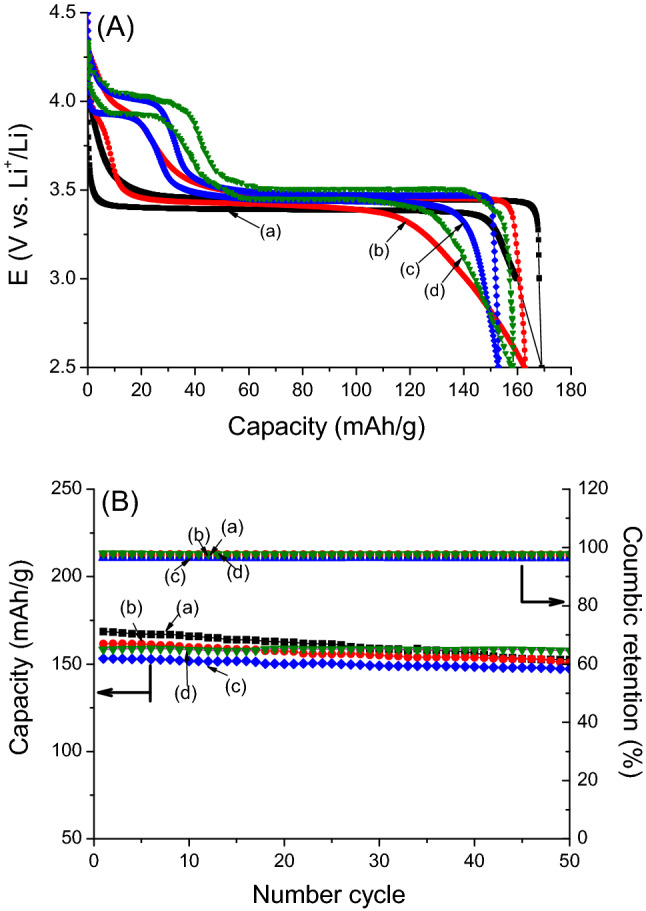
(**A**) Typical charge/discharge curves and (**B**) cycling performance upon 50 cycles of olivine samples at a rate 0.1 C: (a) LiFePO_4_; (b) LiMn_0.1_Fe_0.9_PO_4_; (c) LiMn_0.2_Fe_0.8_PO_4_ and (d) LiMn_0.3_Fe_0.7_PO_4_. These figures have been presented using OriginLab Software, ver.6.0 (https://www.originlab.com).

The galvanostatic cycling tests at a constant current of 0.1 C in the potential range of 2.5–4.3 V are shown in Fig. 5A. It is well known that the LiFePO_4_ profile drops rapidly to plateau at a voltage of 3.39 V when discharging and leaps to 3.45 V when charging. This profile can be considered as a two-phase mechanism between the LiFePO_4_ phase and FePO_4_ phase (Fig. 5A, curve a). However, the Mn substitution in olivine leads a significant change in the cycling profile (Fig. 5A, curve b to curve d). The discharge curve of the lowest Mn-doped sample falls slowly with a light bend over 3.7 V and reaches the main plateau at 3.41 V, while the reverse plateau appears at 3.47 V. It was reasonable that the Mn-redox signal of LiMn_0.1_Fe_0.9_PO_4_ was hardly seen in the obtained voltammogram. In the other Mn-doped samples, the cycling profiles are demonstrated by a well-defined plateau at over 3.45 V and a short one at around 3.90 V. It was recognized that increasing the degree of Mn doping prolongs the capacity in the high voltage region and shortens it in the lower region. Indeed, the capacity was boosted from 20 mAh g^−1^ for LiMn_0.2_Fe_0.8_PO_4_ to nearly 40 mAh g^−1^ for LiMn_0.3_Fe_0.7_PO_4_^27,31^.

On the other hand, it was observed that Mn doping also lead to shifts in the redox potential as well as an expansion of the polarization. Notably, at mid-capacity (~ 75 mAh g^−1^), the discharge potential shifted towards 50 mV for the Fe-redox potential (from 3.40 to 3.45 V) with an increase in the degree of Mn doping and the polarization considerably increased from 50 mV (for LiFePO_4_) to 63 mV (for LiMn_0.3_Fe_0.7_PO_4_). This finding is consistent with the obtained cyclic voltammograms and can be interpreted as (i) the Mn is more electropostitive than Fe and (ii) the Mn substitution of Fe can be expected to strengthen the Fe–O covalence, which raises the Fe^2+^/Fe^3+^ redox energy and shifts the redox voltage of the Fe^2+^/Fe^3+^ couple higher^25^.

The cycling performance of the olivines are presented in Fig. 5B. The initial capacity of LiFePO_4_ reached 169 mAh g^−1^, which was close to the theoretical capacity, however 10% of the initial capacity was lost after 50 cycles with a final capacity of 152 mAh g^−1^. For the Mn-doped olivines, LiMn_x_Fe_1−x_PO_4_ (x = 0.1, 0.2, and 0.3), there was negligible capacity loss and their final capacity reached 151, 147, and 157 mAh g^−1^, respectively. The capacity loss can be interpreted by the release of Mn during the charge–discharge process^41^. The Coulombic retentions were over 95% during the cycling test, which indicated a reversible Li-intercalation into the olivine hosts.

**Figure 6:**
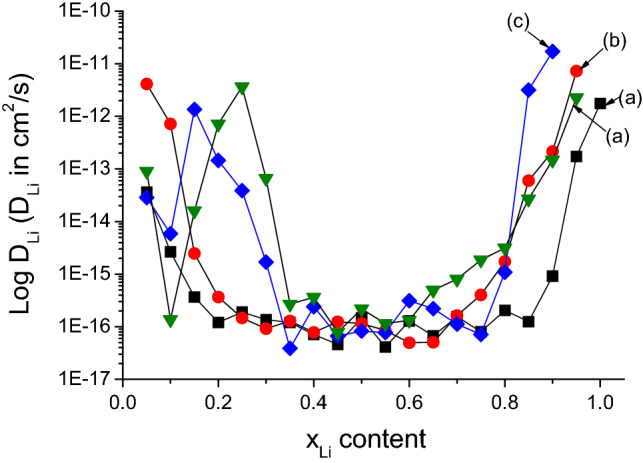
Evolution of D_Li_ as a function of lithium content in olivine samples: (**a**) LiFePO_4_; (**b**) LiMn_0.1_Fe_0.9_PO_4_; (**c**) LiMn_0.2_Fe_0.8_PO_4_ and (**d**) LiMn_0.3_Fe_0.7_PO_4_. These figures have been presented using OriginLab Software, ver.6.0 (https://www.originlab.com).

The apparent chemical coefficient of diffusion of lithium (D_Li_) is a key parameter that evaluates the lithium transport into the intercalation hosts, which can be determined by the galvanostatic intermittent titration technique (GITT). This method imposes a constant current through the cell for a certain time interval^42,43^. The open circuit voltage (OCV) curve was measured with a constant discharge rate of C/50 for 30 min followed by an OCV relaxation period of 5 h to the equilibrium voltage. The diffusion process within the host was assumed to obey Fick’s second law of diffusion, and under galvanostatic conditions, it obeys the following Eq. (2):

2 $$ D = \frac{4}{\pi }\left( {\frac{{V_{M} }}{{S \cdot F \cdot z_{A} }}} \right)^{2} \left[ {I_{0} .\left( {\frac{dE}{{dx}}} \right)/\left( {\frac{dE}{{d\sqrt t }}} \right)} \right]^{2} , $$

where V_M_ is the molar volume of the host, S is the active surface of the electrode, F is Faraday’s constant (F = 96,500 C mol^−1^), z_A_ is the charge of the mobile species (z_Li_ = 1), I_o_ is the magnitude of the current pulse, dE/dx is the slope of the OCV curve, and dE/dt^1/2^ was directly obtained from the measurement of the voltage as a function of time during the constant current flux. The galvanostatic curve described the phase transition between LiFePO_4_–FePO_4_ can be divided in three segments: (i) a quickly dropping voltage as a solid solution segment for Li content below 0.1, (ii) a phase transition segment with Li content ranging from 0.1 to 0.9, and (iii) other solid solution segments for Li content below 1^44,45^. The D_Li_ in the solid solution segment is usually more rapid than those in the phase transition region due to the slope dE/dx. Indeed, the phase transition is characterized by a flat voltage that leads to a small dE/dx. Figure 6 demonstrates the evolution of D_Li_ as a function of lithium content in the olivines. It is observed that the synthesized olivines have a D_Li_ in the range of 10^–17^–10^–15^ cm^2^ s^−1^ at the phase transition with Li content ranging from 0.2 to 0.8 for LiFePO_4_ and LiMn_0.1_Fe_0.9_PO_4_ and from 0.35 to 0.8 for the two other olivines. Moreover, the evolution of D_Li_ in LiMn_0.2_Fe_0.8_PO_4_ and LiMn_0.3_Fe_0.7_PO_4_ shows a peak at x_Li_ = 0.1 due to a short plateau of the redox couple Mn^2+^/Mn^3+^, which is consistent with the galvanostatic curve. Except at the phase transition, the D_Li_ of the Mn-doped olivines were significantly higher than the original olivine, which can be explained by enlarged lattice due to Mn substitution as well as a 1D channel in the (010) plane that is favourable for Li transport. To compare, A. Kumar^20^ has reported a D_Li_ = 1.28 × 10^–15^ cm^2^ s^−1^ and D_Li_ = 7.13 × 10^–14^ cm^2^ s^−1^ for pure LiFePO_4_ and carbon coated LiFePO_4_, respectively. Tang^28^ has also reported that the D_Li_ values were depended onto Li content in LiFePO_4_ olivine in range from 9.0 × 10^−18^ to 4.0 × 10^−14^ cm^2^ s^−1^. Hong^46^ has obtained diffusivity value of 0.70 × 10^−11^ cm^2^ s^−1^ (measured at x = 0.01 for for Li_1−x_FePO_4_) using GITT method. Based on electrochemical impedance spectroscopy (EIS) calculating, Gao^19^ ported a D_Li_ was in the range of 10^−15^–10^−9^ cm^2^ s^−1^ for LiFePO_4_/carbon nanoparticles synthesized by microwave plasma chemical vapor deposition (MPCVD) method. Briefly, these above results on D_Li_ values of LiM_x_Fe_1−x_PO_4_ olivines demonstrated that the D_Li_ values are strongly depended on composition of doped M metals and their content, Li content, particles size of olivines, method for preparation, carbon coating and also calculating methods as well. It can be seen that the obtained D_Li_ values in our results are comparable to previous reports on LiFePO_4_ olivines cathode materials^19,20,28,46,47^, which were determined by electrochemical methods such as CV, EIS, GITT^20,28,48–50^.

## Conclusion

The Mn-doped olivine LiMn_x_Fe_1−x_PO_4_ (x = 0, 0.1, 0.2, and 0.3) were successfully synthesized via the hydrothermal route. Characterization results demonstrated that as synthesized Mn-doped olivines presented an orthorhombic structure with lattice parameters that agreed with prior reports. The electrochemical measurements demonstrate that all Mn-doped olivine samples show better performance than that of original LiFePO_4_. In which of synthesized Mn-doped olivines, the LiMn_0.3_Fe_0.7_PO_4_ exhibits the superior cycling stability with the retention of 100% initial capacity (157 mAh g^−1^) upon 50 cycles. Following the EIS and GITT results, the Mn-substitutions benefits the electron-transfer process as well as the Li-transport in 1D channel (010) plan due to the enlargement of lattice parameter. The olivine LiMn_x_Fe_1−x_PO_4_, therefore, can lead to the development of high-performance lithium-ion batteries.
